# Evaluating the Effect of *Lactobacillus casei* FEGY 9973 and Curcumin on Experimental Giardiasis

**DOI:** 10.1007/s11686-023-00744-4

**Published:** 2023-12-07

**Authors:** Omima M. Abou Shady, Ibrahim Ali Shalash, Fouad M. F. Elshaghabee, Mohamed S. I. Negm, Gehad A. B. Yousef, Enas M. A. Rizk

**Affiliations:** 1 Medical Parasitology Department, Kasr Al-Ainy Faculty of Medicine, Cairo University, Giza, Egypt; 2 Parasitology Department, Theodor Bilharz Research Institute, Giza, Egypt; 3 Department of Dairy Science, Faculty of Agriculture, Cairo University, Giza, Egypt; 4 Pathology Department, Faculty of Medicine, Cairo University, Giza, Egypt

**Keywords:** *Lactobacillus casei*, Probiotic, Curcumin, Giardiasis

## Abstract

**Background and Objective:**

*Giardia* is a parasitic hard protozoan that causes a variety of parasitological and pathological changes in gastrointestinal epithelial cells and is resistant to a variety of disinfectants and treatments. This study used experimental animals infected with *Giardia Lamblia* to assess the potential therapeutic effect of *Lactobacillus casei*, *Lactobacillus bulgaricus* (Lactobacillus in yoghurt) and curcumin in comparison to one of the commonly used drugs (metronidazole).

**Methods:**

The study included 54 Syrian hamsters (*Mesocricetus auratus*) that ranged in weight from 80 to 100 g and were divided into six groups: The effect of the used preparations was assessed in terms of parasitological and histopathological aspects in Group I non-infected healthy control, Group II infected non-treated, Group III infected treated with metronidazole MTZ, Group IV infected treated with *Lactobacillus casei*, Group V infected treated with curcumin, and Group VI infected treated with, *Lactobacillus bulgaricus* (Lactobacillus in yoghurt). The number of *G. lamblia* cysts per gram of stool was counted during the parasitological examination.

**Results:**

The difference between the infected non-treated group and all the treated groups was statistically significant (P0.05). When compared to the infected untreated group, *Lactobacillus casei* and,* Lactobacillus bulgaricus* (Lactobacillus in yoghurt) produced a 100% reduction in *G. lamblia* cyst shedding, curcumin produced an 87.80% reduction in number of cysts, and metronidazole produced a 78.4% reduction in number of cysts.

**Conclusion:**

Our results highlight the potentially effective therapeutic effect of different preparations of probiotics and curcumin against Giardiasis.

## Introduction

Giardiasis is a gastrointestinal parasitic disease caused by the flagellated protozoan *Giardia duodenalis* (also known as *Giardia lamblia* and *Giardia intestinalis*) [1]. *Giardia* is a parasite found in a wide range of mammals, including humans, livestock, pets, wildlife, and aquatic animals, all over the world [2]. Giardiasis is distributed worldwide, but its prevalence varies from 2 to 5% in developed countries to 20 to 30% in developing countries [3]. *Giardia lamblia* was assumed to be a harmless bystander in the microbial gut flora until a few decades ago, but once the parasite’s pathogenicity was discovered, numerous types of medications were utilized to treat it [4]. These medications are mainly from the nitroimidazole, benzimidazole, quinacrine, paromomycin, or furazolidone families; however, due to an increase in the number of emerging resistant cases, new effective alternative treatments are needed [5]. There was an urgent need for alternate bio-therapeutic approaches, which included natural interventions such as probiotics and plant extracts, due to the continued evolution of infections resistant to normal giardiasis treatment, as well as reports of resistant *G. lamblia* strains [6].

When administered in sufficient amounts, probiotics are live microorganisms that benefit the host’s health [7]. Commensal microbiota and *Giardia* trophozoites compete for adhesion sites in the small intestine microenvironment in order to populate it. Different probiotic strains have been shown to reduce the severity and duration of murine giardiasis even while restoring gut morphology; more lately, different *Lactobacillus* species have been discovered to modulate murine giardiasis by preventing *Giardia* trophozoites from adhering to the mucosal surface [8].

*Curcuma longa* (*Zingiberaceae*) is a plant with a long history of therapeutic use in traditional medicine [9].Curcumin is the plant’s main bioactive component, and it has a wide range of pharmacological properties, including antioxidant, anti-inflammatory, antitumor, and antimicrobial properties [10]. Curcumin’s anti-parasitic properties have also gotten a lot of attention in recent decades [11]. Growth, viability, and cell differentiation were all affected. Curcumin also induced a significant reduction in the quantity of trophozoites in intestinal sections and fewer cysts in feces in animal models infected with *G. lamblia* [12].

This study aimed to evaluate the ameliorative therapeutic effect of *lactobacillus casei*, *curcumin* and *Lactobacillus bulgaricus (Lactobacillus in yoghurt)* was assessed in comparison to one of the commercially used drugs (metronidazole) using experimental animals infected with *G. lamblia*.

## Materials and Methods

### The Animal Models

The current study used 54 Syrian hamsters (*Mesocricetus auratus*) that were 5–6 weeks old and weighed around 80–100 g. They were purchased from European Country Farms in Egypt and housed at Theodor Bilharz Research Institute (TBRI). Before the experiment began, they were all free of parasitic infection, as evidenced by a stool examination using the sedimentation technique. The animals were kept in the biological unit of TBRI at a temperature of 24 °C on a regular feed containing 24 percent protein, 4% fat, and roughly 4–5% fiber and water throughout the investigation. Hamster feces, as well as dead hamsters, were examined for hygienic disposal.

### Ethical Approval

This study was performed according to the instructions of the ethical committee of Kasr-Alainy school of Medicine, TBRI and the Cairo University of Institutional Animal Care and Use Committee (CU-IACUC). All the experiments were carried out according to The Clinical and Laboratory Standards Institute (CLSI) guidelines.

### Experiment Design

The animals were divided into six study groups, each with nine animals: Group I (healthy control group with no infection and no treatment), Group II (infected with a single dose of 10,000 *G. lamblia* cyst once without treatment), Group III (infected with* G. lamblia* and treated with metronidazole at a dose of 12 mg/kg/day (1 mg/hamster for 7 days), Group IV (infected with *G. lamblia* and treated with *Lactobacillus casei* FEGY 9973 in a dose of 0.1 mL, Group V (infected with *G. lamblia* and given curcumin in a dose of 20 mg/kg/day (1.6 mg/hamster) single oral dose per day) and Group VI (infected with *G. lamblia* and given, *Lactobacillus bulgaricus* (Lactobacillus in yoghurt) (fermented milk containing *Lactobacillus casei* in a dose of 0.1 mL containing 8.40LogCFU/mL) single oral dose per day *Lactobacillus casei* FEGY 9973 and, *Lactobacillus bulgaricus* (Lactobacillus in yoghurt) and curcumin were given for 30 consecutive days.

### The Infection

Using an esophageal tube, each hamster in the infected groups was infected orally with 1 mL *G. lamblia* cyst suspension containing 10,000 cysts. To avoid traumatic injury to the hamster throat, feces samples were collected weekly after infection and subjected to parasitological investigation using the direct wet mount technique to detect *G. lamblia* cysts. To ensure that all hamsters were infected, the feces of each hamster must be separated.

### The Drug Administration

Three weeks post-infection, *G. lamblia* cysts were detected in stool with more than 8 cyst in the field indicating heavy infection of hamsters and the treatment was initiated. According to Taha *et al.* [13], the drug dose was given in mg/kg (=*x*) and was adjusted to hamster weight via the following formula:

$$x = \frac{{\left( {{\text{Average human dose \,in}}\,\frac{{{\text{mg}}}}{{{\text{kg}}}} \times {\text{ average hamster weight in kg }}} \right)}}{{\text{Average human weight in kg}}}$$

*Lactobacillus casei* FEGY 9973 was grown on MRS by incubation at 37 °C for 18 h in the Department of Dairy Science, Faculty of Agriculture, and Cairo University, Egypt. According to Hassan and Elshaghabee [14], fermented milk with *L. casei* FEGY 9973 was prepared. Using MRS agar (de Man Rogosa Sharpe medium acquired from Hi-media Laboratories agar) at 37 °C for 72 h, aerobic, the viable count of *L. casei* strain in fermented milk samples was 8.40 Log CFU/mL. Every 5 days, subcultures were performed by mixing 0.2 ml mother culture with 10 ml sterile milk and incubated for 24 h at 37 °C. Curcumin powder is dissolved in water at a ratio of 1:10.

### Statistical Analysis

For statistical analysis, data were coded and inputted using the statistical package software SPSS model 23 (Chicago, IL, USA). Data have been tabulated, and descriptive statistics for quantitative variables have been defined using mean, range and the standard deviation, while descriptive statistics for qualitative variables have been defined using frequency and percentage. Data were considered statistically significant if the *P* value was < 0.05.

## Results

### Parasitological Assessment of the Therapeutic Effect of the Tested Preparations

Cysts counting of all groups continued for 30 days of treatment till the end of the experiment. The cysts’ count was performed in the (3rd, 5th, 7th, 11th, 17th, 19th, 21st, 23rd, 25th, 30th) day of treatment. It was noticed that there was a continuous reduction in the cyst count throughout the study until the end but there were a significant difference between used preparations, on 3rd day of treatment a reduction in the cyst count began to be noticed as the number of *G. lamblia* cysts/gm of feces that were shedded. The reduction in cysts shedding in Groups III, IV, V, and VI had a statistical significant difference compared to Group II (infection control group) (*P* value < 0.05) and the reduction in cysts shedding in Groups IV, V, and VI had a statistical significant difference compared to Group III (infected treated with metronidazole) (*P* value < 0.05) (Table 1).

**Table 1:** Comparison between infected untreated group and infected treated groups regarding number of cysts (mean ± SD)/g feces and percentage of reduction at 3rd day of treatment

	Number of cysts/g at 3rd day	Reduction (%)	*P* value
Group II	37.00 ± 9.11 × 10^3^		< 0.05
Group III	18.8 ± 1.92 × 10^3^	49.18	
Group IV	16.04 ± 2.07 × 10^3^	55.6	
Group V	25.00 ± 5.61 × 10^3^	32.43	
Group VI	17.6 ± 2.30 × 10^3^	52.43	

Reduction in shedding of *G. lamblia* cyst continued throughout the experiment and was recorded every other day in the (3rd, 5th, 7th, 11th, 17th, 19th, 21st, 23rd, 25th, 30th) day of treatment, it was observed that groups treated with *Lactobacillus casei* and *Lactobacillus bulgaricus* (Lactobacillus in yoghurt) showed the best results with the highest reduction rate up to 100% on day 25 with complete eradication of *G. lamblia* cyst from hamster feces, while group treated with curcumin showed 87.80% reduction in the percentage of *G. lamblia* cyst at the end of the experiment. However, the group treated with metronidazole had 78.4% reduction rate at the end of the experiment (Table 2, Fig. 1).

**Table 2:** Comparison between cysts shedding/g feces × 10^3^ in different days of treatment in different groups

	3rd	5th	7th	11th	17th	19th	21st	23rd	25th
Group II	37.00 ± 9.11	77 ± 29.33	138.8 ± 10.13	272.33 ± 13.81	133 ± 13.52	88.33 ± 5.50	64. ± 3.83	37.86 ± 13.1	15.0 ± 7.91
Group III	18.8 ± 1.92*	41.67 ± 7.69*	53.4 ± 2.07*	59.76 ± 4.36*	30 ± 1 0.00*	26.5 ± 1.87*	22 ± 1.58*	10.0 ± 3.74*	3.25 ± 1.26*
Group IV	16.04 ± 2.07*#	28.5 ± 4.76*#	42.2 ± 3.7*#	44.67 ± 4.89*#	20.00 ± 1.58*#	10.86 ± 5.08*#	2.00 ± 1.58*#	0.33 ± 0.52*#	00.00*
Group V	25.00 ± 5.61 *#	64.50 ± 16.66*#	88.60 ± 2.70*#	152.50 ± 9.35#*	45.00 ± 9.85*#	30.14 ± 3.34*#	15.2 ± 2.39 *	5.83 ± 2.48*#	2.50 ± 0.58*
Group VI	17.6 ± 2.30*#	30.67 ± 4.72 *#	43 ± 3.39*#	45.17 ± 4.36*#	22.00 ± 1.58*#	15.29 ± 3.25*#	4.20 ± 3.49*#	2 ± 1.41*#	00.00*

Values are expressed as mean ± SD

*Statistically significant compared to infection control group = *P* value < 0.05

#Statistically significant compared to metronidazole-treated group = *P* value < 0.05

**Fig. 1:**
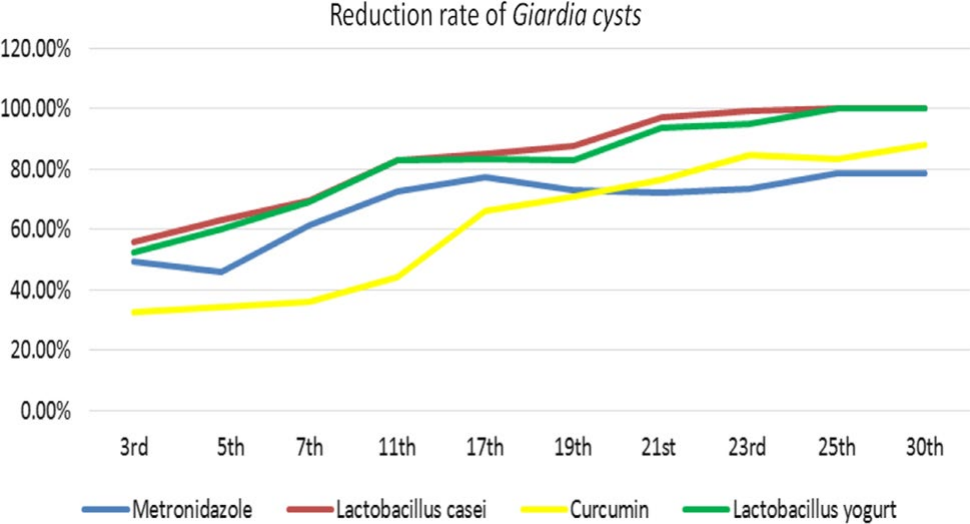
Reduction rate of *G. lamblia* cysts throughout treatment in all groups

### The Therapeutic Efficacy of the Tested Preparations was Examined Histopathologically

**Fig. 2:**
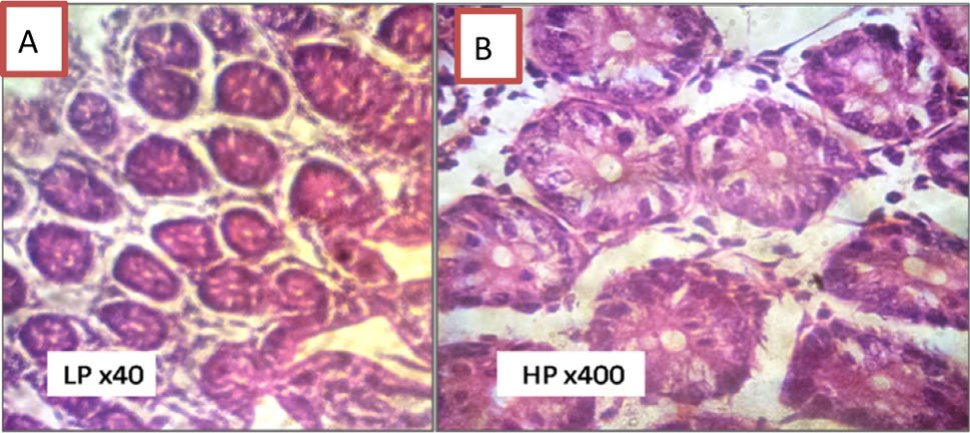
Transverse section of normal hamster’s duodenum found in negative control group in the form of normal villous architecture and moderate number of goblet cells

In Group I, histopathological examination revealed normal duodenal mucosa in the form of normal villous architecture with average villi length and width, a moderate amount of goblet cells, and a well-defined brush border (Fig. 2).

**Fig. 3:**
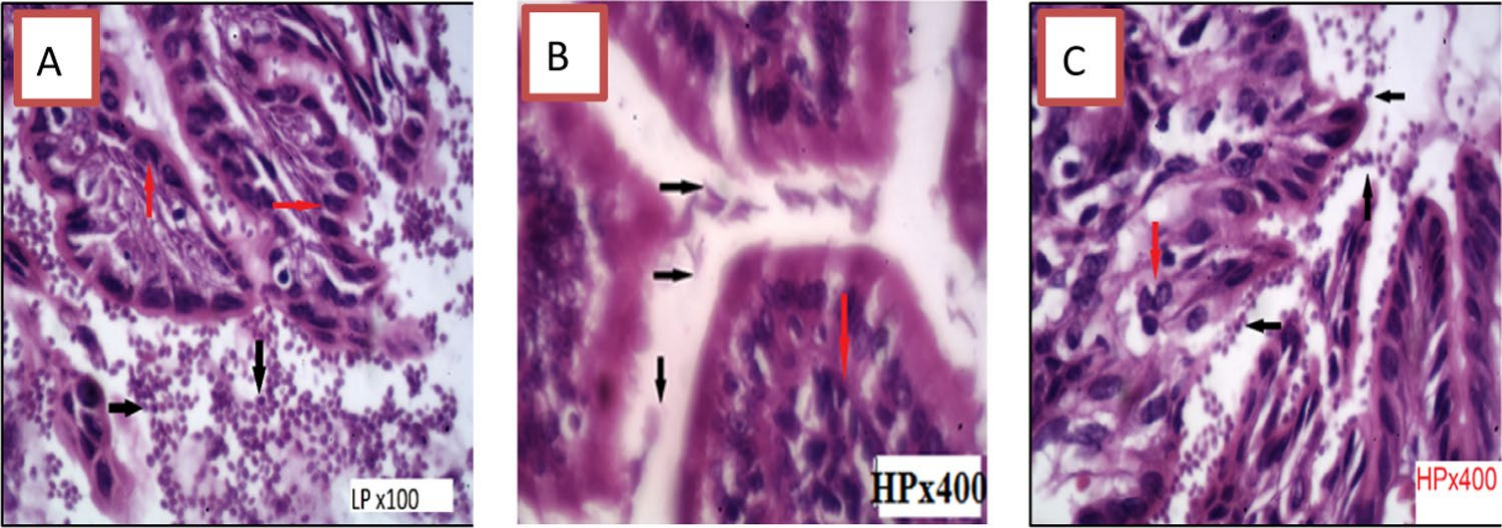
Transverse section of hamster’s duodenum showing severe active inflammation found in infected untreated Group II, red arrow showing lymphocytic aggregation, black arrow showing large number of *G. lamblia* trophozoite attached to brush border of the villi and in intestinal lumen

The infected group (Group II) had significant active duodenitis, as evidenced by villi shortening and boarding. *G. lamblia*, trophozoites appeared as pear-shaped bodies, colorless to pale pink with darker nucleus, attached by ventral disc to the brush border of the epithelia, lamina propria showed edema and infiltration with inflammatory cells; mainly plasma cells, lymphocytes, and large number of neutrophils with diffuse loss of brush border microvillus surface area, *G. lamblia*, trophozoites appeared as pear-shaped bodies, colorless (Fig. 3).

**Fig. 4:**
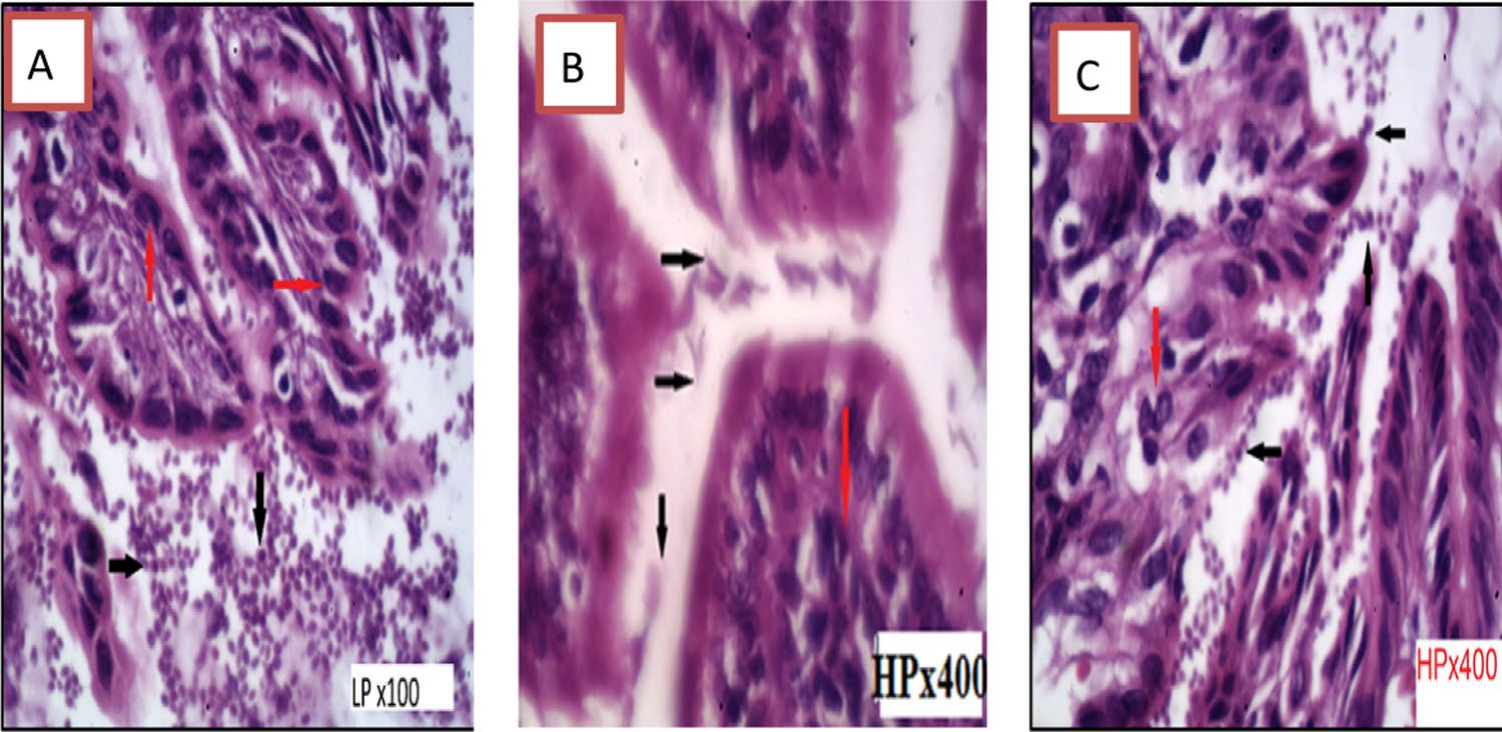
Transverse section of hamster’s duodenum showing moderate active inflammation with edematous villi with core infiltration by inflammatory cells and lymphocytic aggregation found in the infected treated group with metronidazole. Red arrow showing lymphocytic aggregation

**Fig. 5:**
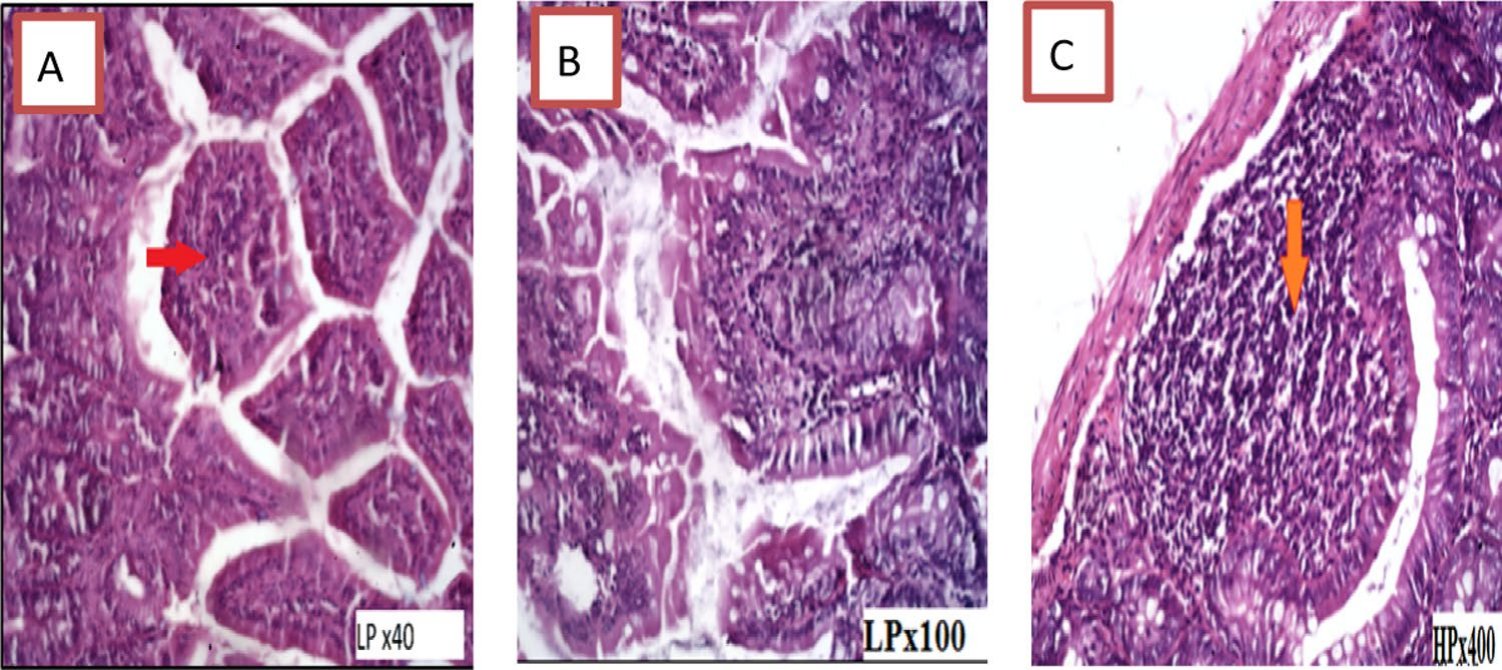
Transverse section of hamster’s duodenum showing mild duodenitis. Mild core cellular infiltration of lamina propria found in the infected groups treated with lactobacillus casei and lactobacillus yogurt. Arrow showing cellular infiltration

**Fig. 6:**
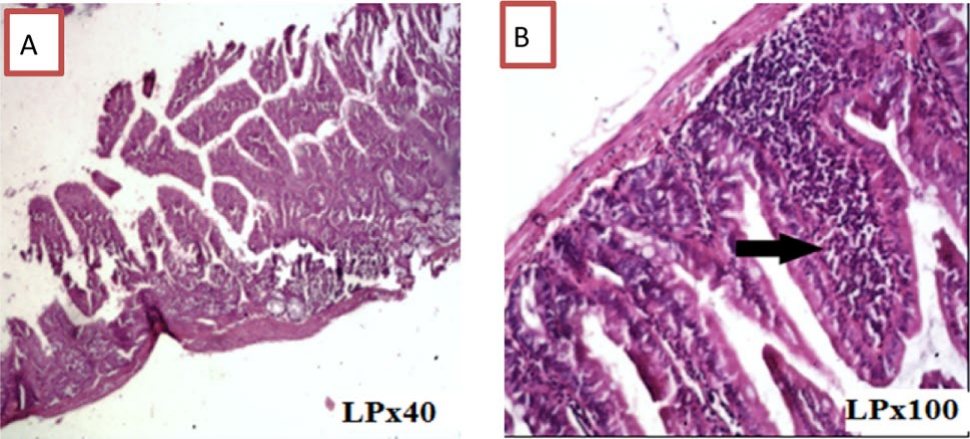
Transverse section of hamster’s duodenum showing mild active inflammation with cellular infiltration found in the infected groups treated with curcumin. Arrow showing inflammatory cellular infiltration

In Group III, histopathological examination of sections from the duodenum revealed moderate active duodenitis in the form of loss of villous architecture with shortening and boarding of villi and edema of the lamina propria with moderate inflammatory cellular infiltrate including plasma cells and lymphocytes and a moderate number of neutrophils, whereas Group IV showed remarkable improvement with mild duodenitis in the form of preserved villous configuration and mild edema. In Group V, the studied duodenal sections exhibited mild active duodenitis in the form of maintained villous shape and mild lamina propria edema, as well as a mild mixed inflammatory cellular infiltrate involving plasma cells, lymphocytes, and scattered neutrophils. Mild duodenitis with preserved villous shape and mild edema of the lamina propria were found in pancreatic duodenal slices from Group VI, as well as a mild inflammatory cellular infiltrate involving plasma cells and lymphocytes (Figs. 4, 5, 6).

## Discussion

*G. lamblia* is a widespread fecal–oral parasite of the small intestine that is one of the leading causes of diarrhea in humans and animals around the world [15]. Probiotics may prevent *G. lamblia* infection through a variety of mechanisms, including competition for limited adhesion sites, competition for nutrients that *G. lamblia* would otherwise consume, stimulation of the host immune response, and the production of substances that may inhibit *G. lamblia* [16, 17]. Probiotic substances, mostly generated from *Lactobacilli*, have been shown to have anti-*G. lamblia* action [17]. Plants that are used to treat gastrointestinal disorders including diarrhea and dysentery have increased the potential for new alternative therapies [18]. The anti-giardiasis effect of four regimens was tested in experimentally infected hamsters. Assessment of anti-giardial activity of each treatment model was conducted by performing parasitological examination by evaluating the number of cysts, as well as histopathological examination by evaluating the mucosal integrity and submucosal cellular infiltrates in duodenal biopsies.

In our study, probiotic *Lactobacillus* caused a significant reduction in the number of *G. lamblia* cysts, possibly due to its anti-giardial effect via the production of antimicrobial compounds and competition for nutrients, as well as interfering with *G. lamblia* trophozoite attachment to the mucosa. It also confers host health benefits by enhancing innate and adaptive immune responses [19].* Lactobacillus johnsonii* La1, *Lactobacillus casei* MTCC1423, and *Lactobacillus rhamnosus* GG (LGG), for example, have been demonstrated to exhibit anti-giardial characteristics by inhibiting the development of trophozoites and lowering the severity of infection in different mouse models [20].

In the current investigation, the curcumin effect demonstrated that at a dose of 20 mg/kg/day, it reduced the number of cysts by 87.80% as compared to the infected untreated group. The findings of this study match with those of Dyab *et al.* [11] who found an 84.7% reduction in cyst count in infected mice treated with curcumin at a dose of 20 mg/kg/day for 7 days. Curcumin suppresses trophozoite attachment and growth, according to Said *et al.* [21], resulting in a significant reduction in the quantity of trophozoites in intestinal sections and cysts in feces. It damages trophozoites by causing changes to their dorsal and ventral surfaces. Damage to membrane blebs, a change in the lateral flange, damage to the caudal area, ventral disc, and flagella, and a loss of normal morphology are all consequences. Curcumin also affects *G. lamblia* cytoskeletal structures, showing that it serves as a giardial microtubule destabilizer, according to Filiberto *et al.* [12]. In the current study, duodenum sections from Group V showed mild duodenitis in the form of preserved villous configuration and mild edema of the lamina propria with mild mixed inflammatory cellular infiltrate including plasma cells, which is consistent with Dyab *et al.* [11] research results of noticeable improved performance of the pathological changes of the villous architecture following ginger and curcumin treatment.

In the current study, Metronidazole (MTZ) resulted in a 78.40% percent reduction in the number of *G. lamblia* cysts. Renata *et al.* [22] reported that Metronidazole, used as a single drug and administered only once daily for 7 days, resulted in a decrease in the elimination of *G. lamblia* cysts in the feces in 80% of animals, turning from high to low parasitic load (less In a study comparing the therapeutic effects of lauric acid against MTZ on *G. lamblia* in experimentally infected hamsters, Aly *et al.* [23] reported a 93.77% reduction in the number of cysts when MTZ was used. While treatment with Metronidazole MTZ improved pathological findings in the current investigation when compared to the infected untreated group, Aly *et al.*, [23] showed partial repair of the intestinal mucosa with focal flattening of surface enterocytes in the MTZ-treated group.

## Conclusion

The current study found that *Lactobacillus casei*, *Lactobacillus bulgaricus* (Lactobacillus in yoghurt), and curcumin had an anti-giardiasis effect in *G. lamblia*-infected hamsters, with a higher percentage of reduction than metronidazole. More trials with these other strains of *Lactobacillus* and curcumin at different doses are needed to find the most effective treatment for giardiasis.
